# Maternal Anxiety and Infants Birthweight and Length of Gestation. A sibling design

**DOI:** 10.1186/s12888-021-03620-5

**Published:** 2021-12-07

**Authors:** Mona Bekkhus, Yunsung Lee, Ragnhild Eek Brandlistuen, Sven Ove Samuelsen, Per Magnus

**Affiliations:** 1 Promenta Research Centre, Department of Psychology, University of Oslo, PB 1094 Blindern, 0317 Oslo, Norway; 2 Centre for Fertility and Health, Norwegian Institute of Public Health, Oslo, Norway; 3 Department of Genetics and Bioinformatics, Norwegian Institute of Public Health, Oslo, Norway; 4 Department of Child Health and Development, Norwegian Institute of Public Health, Oslo, Norway; 5 Department of Mathematics, University of Oslo, Oslo, Norway

**Keywords:** Birthweight, Gestational age, Prenatal maternal anxiety, Sibling study

## Abstract

**Background:**

The overall aim of this study is to examine the effect of prenatal maternal anxiety on birthweight and gestational age, controlling for shared family confounding using a sibling comparison design.

**Methods:**

The data on 77,970 mothers and their 91,165 children from the population-based Mother, Father and Child Cohort Study and data on 12,480 pairs of siblings were used in this study. The mothers filled out questionnaires for each unique pregnancy, at 17^th^ and 30^th^ week in pregnancy. Gestational age and birth weight was extracted from the Medical Birth Registry of Norway (MBRN). Associations between prenatal maternal anxiety (measured across the 17^th^ and 30^th^ weeks) and birth outcomes (birthweight and gestational age) were examined using linear regression with adjustment for shared-family confounding in a sibling comparison design.

**Results:**

In the population level analysis the maternal anxiety score during pregnancy was inversely associated with new-born’s birthweight (Beta = -63.8 95% CI: -92.6, -35.0) and gestational age (Beta = -1.52, 95% CI: -2.15, -0.89) after adjustment for several covariates. The association of the maternal anxiety score with birthweight was no longer significant, but remained for maternal anxiety at 30^th^ week with gestational age (Beta = -1.11, 95% CI: -1.82, -0.4) after further adjusting for the shared-family confounding in the sibling comparison design.

**Conclusion:**

No association was found for maternal prenatal anxiety with birth weight after multiple covariates and family environment were controlled. However, there was an association between prenatal maternal anxiety at 30^th^ week only with gestational age, suggesting a timing effect for maternal anxiety in third trimester.

## Introduction

Preterm birth (PTB) and low birthweight (LBW) are related to perinatal mortality, and have been associated with a wide range of adverse developmental outcomes for children [1]. Recently, much focus has been on the potential impact of maternal distress and anxiety, such as feeling fearful or nervous on birth outcomes. Studies have reported that the prevalence of maternal anxiety during pregnancy varies from 6.6 to 10.4% [2, 3]. Further, maternal anxiety during pregnancy is associated with birthweight and gestational age [4, 5]. Prenatal anxiety is recognized as a potential risk factor for PTB and LBW [6–8].

While associations between prenatal maternal anxiety and PTB and LBW have been reported, causal inferences are only tentative. Addressing residual confounding by randomizing pregnant mothers to the exposure is clearly not possible [9]. Thus, in humans, investigations are limited to observational designs, and new approaches are needed to advance the field by adequately controlling for genetic and social confounding [9, 10].

In this study, we estimate the effects of degree (no symptoms, light symptoms and severe symptoms) and timing of prenatal maternal anxiety on infant birthweight and preterm birth. Timing was accounted for by examining whether symptoms were reported at either or both of the 17^th^ or 30^th^ gestational weeks. We test these associations using a sibling comparison design. The design, which involves studying birth outcomes following differential exposure to anxiety during pregnancy across siblings, has been found to reduce the extent of the impact of family-level confounding factors [9–11], on examinations of prenatal anxiety effects.

## Methods

**Fig. 1:**
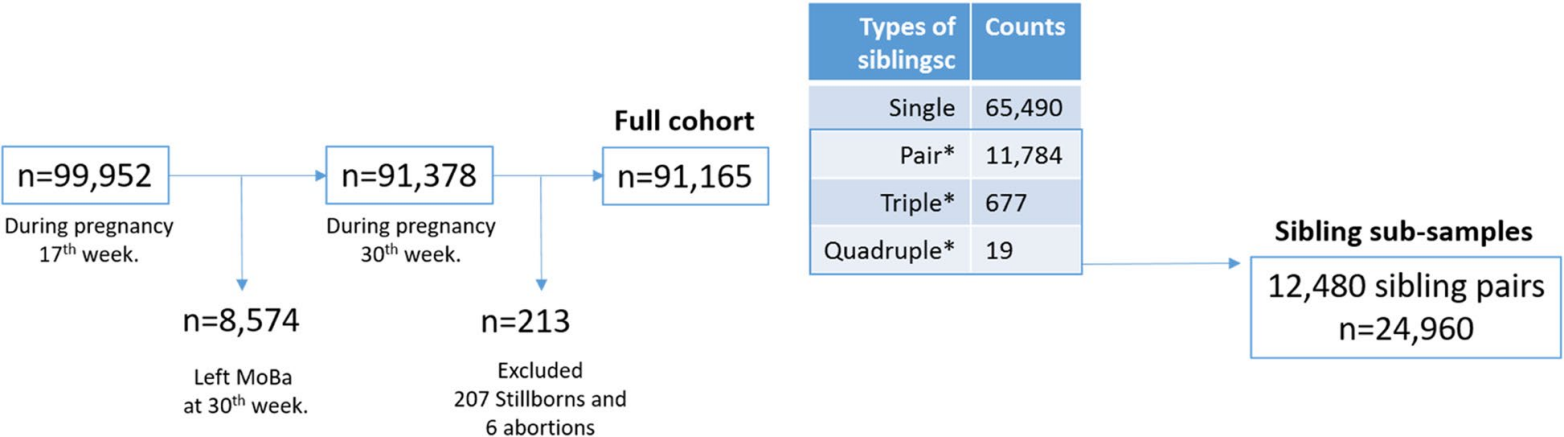
Flow chart for the study population. Note: 8,574 women did not answer the second questionnaire. Two siblings were randomly selected from these types of siblings (*)

In The Norwegian Mother, Father and Child Cohort study (MoBa) [12], participants were recruited from all over Norway from 1999-2008, when attending routine ultrasound -examinations. The women consented to participation in 41% of the pregnancies, and 78,117 mothers of 91,378 children completed questionnaires at the 17^th^ and 30^th^ week of gestation of each unique pregnancy (Fig. 1). Women were included if they responded to these questionnaires, and had available data in the Medical Birth Registry of Norway (MBRN).

The questionnaires asked the mothers to report their anxiety level, in addition to providing information regarding their age, education, marital status, smoking and drinking habits, and their relationship with their partner. The MoBa cohort [12] was also linked to the MBRN, which contains detailed medical information about new-born children (gender, birthweight, and gestational age) as well as their mothers (parity and birth complications).

Among these full data sets, there were 12,480 pairs of siblings. The mean age difference between siblings was 2.77 years. The numbers of pairs dropped slightly after excluding half-siblings who had the same mother but different fathers (Fig. 1). We used version eight of the quality-assured data files. The establishment of MoBa and initial data collection was based on a license from the Norwegian Data Protection Agency and approval from The Regional Committees for Medical and Health Research Ethics (REK- 2009/1899-7). The MoBa cohort is now based on regulations related to the Norwegian Health Registry Act. The current study was approved by The Regional Committees for Medical and Health Research Ethics (REK 2016/1424).

## Measures

### Measures of maternal general anxiety

Mothers reported symptoms of anxiety using validated short versions of two Hopkins Symptom Checklists, the SCL-5 (at 17^th^ week of gestation) and SCL-8 (at 30^th^ week of gestation). Participants answered to the question ‘Have you been bothered by any of the following during the last two weeks’. Items, reflecting e.g. feeling fearful, nervousness or shakiness inside were scored on a Likert scale ranging from 1 (not at all bothered) to 4 (very much bothered) and have been validated at a correlation of 0.92 with the SCL-25 [13]. Assessments of anxiety were reported in the 17^th^ week by two items from SCL-5, and four questions from SCL-8 in the 30^th^ week of each pregnancy. The mean score for the 17^th^ and 30^th^ gestational week measure ranged from 1-4. The “week 17” in Tables 3 and 4 represents the effect of maternal anxiety score at 17^th^ gestational week on the birth outcomes (birthweight and gestational age), and so does the “week 30” that of maternal anxiety score at 30^th^ week on the birth outcomes. The “Both” represents the additive effect of maternal anxiety score at 17^th^, 30^th^ gestational week and the interaction.

The mean score for the two assessments ranged from 1.2 to 1.4 and included the following items: constantly frightened or anxious; nervous, inner turmoil; tense or stressed; and sudden fear without reason.

### Birthweight and Gestational age

Birthweight (measured in grams) was extracted from the Medical Birth Registry of Norway (MBRN) and was treated as a continuous scale producing a mean value of 3,608 (SD 544.8). Gestational age (measured in days) was also treated as a continuous variable and the mean score was 39.5 (SD 1.72). All maternity units in Norway must notify the MBRN of all births and pregnancies terminating after week 12. The MBRN includes information on pre- and post-pregnancy variables including medication, birth complication, and maternal complications.

### Assessment of potential confounders

Potential confounding factors were considered based on whether they could influence both prenatal maternal anxiety and child development outcomes, and were included in the adjusted model if associated with the exposure (prenatal maternal anxiety) and one of the two outcome measures. The following variables were considered to be potential confounders: alcohol consumption during pregnancy (coded as ‘0’ for never and ‘1’ for more than once a month); smoking in pregnancy (coded as ‘0’ for never, ‘1’ for sometimes, and ‘2’ for daily); marital status (coded as ‘0’ for married/living together and ‘1’ for single); and maternal education coded as ‘0’ for higher university degree (+4 years college/university), and ‘1’ (3 years college/university), ‘2’ for 1-2 years college, and ‘3’ for secondary school). The following variables extracted from the MBRN were also controlled for: maternal age as a continuous variable; parity coded as itself, where greater than or equal to 4 was coded as 4+; birth complications (coded as ‘1’ for yes or ‘0’ for no); child’s sex (coded as ‘0’ for girl and ‘1’ for boy); and preeclampsia (coded as ‘0’ for no and ‘1’ for yes).

### Statistical analyses

Multiple regressions were used to estimate the effects of prenatal maternal anxiety on birthweight and gestational age. In a full cohort where all subjects with the exposures, outcomes and adjusting variables were included, we regressed birthweight and gestational age on maternal anxiety score at 17^th^ and 30^th^ gestational week, their interactions (Crude in Table 3 and 4), and further included adjusting variables step by step. Step1 encompassed maternal smoking and alcohol intake, and step 2 included in addition parity, birth complication, child’s sex and preeclampsia. Step 3 added maternal age, education and marital status. Regression analyses were performed in R 4.0.0.

In the sibling comparison design, we selected all available sibling pairs (for families participating with three or more children, one sibling pair was randomly selected) and computed the difference values in each variable between a sibling and the other. Similar to the analyses in the full cohort, we regressed the sibling-difference values in the outcomes on those in the exposures, controlling for family shared effects. Here, the three steps of adjustments were made in the same manner as in the full cohort.

We performed multiple imputations to handle the missing data points in the exposures, outcomes and adjusting variables. The mice R package was used; the number of imputations was set to be 5 (m=5), and the imputation method in use was predictive mean matching (method=“pmm”).

## Results

In the full cohort (Table 1), the mean birthweight of infants of mothers reporting severe anxiety symptoms in the 17^th^ gestational week was 85 grams lower than that of infants born to mothers with very light anxiety symptoms or none at all (*p*<0.001). Similarly, the mean birthweight of infants born to mothers reporting severe anxiety symptoms in the 30^th^ gestational week was 95 grams lower than that of infants not exposed to high levels of maternal anxiety (p<0.001). The mean gestational age between light and severe maternal anxiety differed by 2.2 days for those reporting maternal anxiety in the 17^th^ gestational week (*p*<0.001). Between light and severe anxiety symptoms reported in the 30^th^ gestational week, the mean gestational age decreased by 3.4 days (*p*<0.001).

**Table 1:** Characteristics of full cohort

	Count	Birthweight (in grams)		Gestational age (in days)	
		Mean	SD	Mean	SD
	n=91,165	μ=3,610.3	s=542.3	μ=279.7	s=11.7
Maternal anxiety at week 17 (score)					
1-2, Light	80,233	3,615.1	540.2	279.8	11.7
2-3	8,022	3,581.1	554.3	279.1	12.1
3-4, Severe	1,362	3,529.9	573.0	277.6	12.9
NA	1,548	3,583.5	545.7	279.0	12.0
Maternal anxiety at week 30 (score)					
1-2, Light	85,064	3,615.0	537.3	279.9	11.5
2-3	4,690	3,559.4	572.9	278.5	13.2
3-4, Severe	639	3,519.8	643.1	276.5	15.6
NA	772	3,470.6	728.9	275.5	20.1
Maternal age					
<25	9,862	3,552.4	547.6	279.0	12.3
25-29	30,223	3,592.8	533.2	279.7	11.6
30-34	35,280	3,631.6	536.4	279.9	11.4
>=35	15,800	3,632.0	564.9	279.6	12.2
Partner harmony (score)					
1-2, Good	64,618	3,602.6	540.2	279.8	11.8
2-3	19,726	3,638.9	538.4	279.8	11.4
3-4	3,977	3,646.6	537.8	279.6	11.2
4-5	919	3,637.1	581.1	278.8	12.1
5-6, Bad	359	3,518.4	570.4	278.8	11.5
NA	1,566	3,476.8	617.3	278.0	14.8
Education					
University 4y+	20,406	3,602.9	525.4	280.3	11.3
College/University 3y	35,732	3,620.1	534.3	279.8	11.6
College 1-2y	12,684	3,623.4	555.7	279.7	12.0
Secondary school	17,720	3,595.2	566.3	278.8	12.3
NA	4,623	3,588.3	542.6	279.8	12.0
Marital status					
Married/Partner	87,656	3,613.7	540.9	279.7	11.7
Single	3,509	3,523.1	568.3	279.1	12.3
Parity					
0	41,316	3,505.5	535.4	279.9	12.5
1	32,276	3,683.5	523.1	279.8	10.8
2	13,754	3,725.8	541.4	279.4	11.1
3	2,912	3,723.1	562.3	278.7	11.8
4	907	3,661.1	601.8	276.5	13.1
Alcohol consumption during pregnancy					
Never	74,786	3,611.1	541.7	279.7	11.8
1+/month	533	3,567.4	537.9	280.4	12.1
NA	15,846	3,607.6	545.1	279.8	11.7
Smoking during pregnancy					
None	81,879	3,621.6	538.9	279.8	11.6
Sometimes	4,018	3,570.9	554.5	279.9	12.3
Daily	3,746	3,420.1	553.4	278.5	12.4
NA	1,522	3,571.3	566.9	279.2	13.2
Birth complication					
No	66,220	3,627.8	546.0	280.1	11.9
Yes	24,945	3,563.6	529.3	278.6	11.3
Child sex					
Girl	44,450	3,546.2	524.6	279.2	11.5
Boy	46,715	3,671.2	551.7	280.2	12.0
NA	87,792	3,624.7	524.6	280.1	11.2
Preeclampsia					
Yes	3,373	3,234.0	799.2	270.2	19.2
No	80,233	3,615.1	540.2	279.8	11.7

Among the 24,960 siblings (see Table 2), a mean birthweight difference between infants born to mothers reporting light anxiety symptoms and those born to mothers reporting severe anxiety symptoms was also found. However, for the sibling subsample, only a 44 gram difference in mean birthweight was recorded for infants exposed to light anxiety or none at all, as reported in the 17^th^ gestational week (p<0.01). Mean birthweight for infants exposed to severe compared to light or no maternal anxiety, as reported in the 30^th^ gestational week, differed by 153 grams (p<0.01). Mean gestational age decreased by 1.4 days between infants born to mothers reporting severe symptoms and infants born to mothers reporting light symptoms in the 17^th^ gestational week (p<0.05); however, this difference was 3.5 days for those reporting symptoms in the 30^th^ gestational week (p<0.001).

**Table 2:** Characteristics of sibling sub-sample

	Count	Birthweight (in grams)		Gestational age (in days)	
		Mean	SD	Mean	SD
	n=24,960	μ=3,639.5	s=523.0	μ=280.0	s=11.3
Maternal anxiety at week 17 (score)					
1-2, Light	22,888	3,642.9	521.4	280.1	11.2
2-3	1,571	3,602.7	538.5	279.5	11.8
3-4, Severe	219	3,598.8	510.1	278.7	11.0
NA	282	3,596.6	563.2	279.1	12.0
Maternal anxiety at week 30 (score)					
1-2, Light	23,836	3,642.5	519.2	280.1	11.1
2-3	890	3,605.8	562.6	279.3	12.8
3-4, Severe	104	3,489.5	575.4	276.6	14.6
NA	130	3,444.3	767.5	274.0	22.3
Maternal age					
<25	2,306	3,598.8	540.8	279.1	12.0
25-29	8,883	3,623.9	521.0	280.0	11.3
30-34	10,265	3,655.6	517.1	280.2	11.0
>=35	3,506	3,658.4	530.4	280.1	11.4
Partner harmony (score)					
1-2, Good	18,030	3,628.7	522.6	280.0	11.3
2-3	5,497	3,669.9	516.2	280.3	10.9
3-4	984	3,686.4	518.7	280.1	10.5
4-5	198	3,698.8	549.8	278.6	13.0
5-6, Bad	47	3,577.9	470.7	280.7	9.4
NA	204	3,501.4	660.7	276.8	16.8
Education					
University 4y+	6,432	3,634.1	507.0	280.5	10.9
College/University 3y	10,958	3,641.6	523.6	280.0	11.2
College 1-2y	2,945	3,647.4	528.8	279.8	11.7
Secondary school	3,572	3,635.8	541.7	279.3	11.6
NA	1,053	3,640.2	531.7	280.5	11.4
Marital status					
Married/Partner	24,420	3,640.4	522.3	280.0	11.3
Single	540	3,596.7	548.9	278.9	11.4
Parity					
0	9,425	3,535.0	528.3	280.1	12.4
1	11,388	3,692.1	505.2	280.1	10.3
2	3,279	3,739.1	513.3	279.8	10.5
3	654	3,736.3	514.2	279.5	11.2
4	214	3,619.2	616.9	276.6	13.5
Alcohol consumption during pregnancy					
Never	21,072	3,641.0	521.0	280.0	11.2
1+/month	97	3,602.8	559.2	281.5	13.5
NA	3,791	3,632.0	532.8	280.0	11.5
Smoking during pregnancy					
None	23,251	3,646.5	521.3	280.0	11.2
Sometimes	743	3,577.1	533.9	280.4	11.7
Daily	589	3,442.7	528.1	278.6	12.0
NA	377	3,637.3	529.2	279.9	13.0
Birth complication					
No	17,519	3,662.7	528.6	280.5	11.5
Yes	7,441	3,584.8	505.3	279.0	10.7
Child sex					
Girl	12,172	3,579.4	504.2	279.5	10.9
Boy	12,788	3,696.7	534.0	280.5	11.6
NA	24,164	3,650.9	507.5	280.3	10.8
Preeclampsia					
Yes	796	3,292.1	796.2	270.8	18.8
No	22,888	3,642.9	521.4	280.1	11.2

The differences in maternal anxiety levels between two pregnancies were also examined. Of the mothers who participated MoBa twice, 4,015 mothers (32%) experienced more anxiety during their first pregnancy than during their second pregnancy, while 2,252 mothers (18%) had the opposite experience. The rest of the mothers (50%) experienced the same anxiety levels during their first and second pregnancies. The mean difference between maternal anxiety during first and second pregnancies was small (mean_diff_ = 0.05).

### Regression analyses for prenatal maternal anxiety and birthweight

**Fig. 2:**
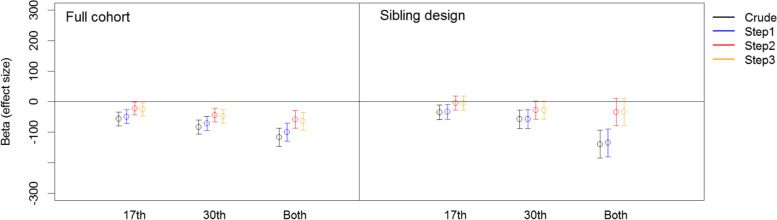
Effect of maternal anxiety on birth weight. Crude: no adjustment; Step1: adjusted for smoking and alcohol intake; Step2: Step1 + parity, birth complication, child’s sex and preeclampsia; Step3: Step1 + Step2 + maternal age, education and marital status

First, as shown in Table 3 (and Fig. 2), the maternal anxiety score at the 17^th^ week was associated with birthweight. A stronger association was found for those reporting symptoms in the 30^th^ week. Children exposed to maternal anxiety prenatally, as reported at both the 17^th^ and 30^th^ gestational weeks, were at higher risk of low birthweight compared to those not exposed and those exposed only once during pregnancy. This association was reduced, but remained, after adjusting for smoking and alcohol consumption. In addition, adjustments for potential confounders at steps 2 and 3 found the association to be weaker but still significant.

**Table 3:** Effect of maternal anxiety on birth weight

	Crude	Step1^*^	Step2^**^	Step3^***^
	Beta [95% CI]	Beta [95% CI]	Beta [95% CI]	Beta [95% CI]
**Full cohort**				
No Anxiety	0 [Reference]	0 [Reference]	0 [Reference]	0 [Reference]
Week 17	**-56.5 [-78.8, -34.2]**	**-48.9 [-71.2, -26.6]**	**-22 [-43.7, -0.4]**	**-25.4 [-47.1, -3.8]**
Week 30	**-83.1 [-106.7, -59.5]**	**-71.4 [-94.9, -47.8]**	**-44 [-66.7, -21.2]**	**-47.8 [-70.6, -25.1]**
Both	**-116.6 [-146.3, -86.9]**	**-99.2 [-128.8, -69.5]**	**-58 [-86.8, -29.2]**	**-63.8 [-92.6, -35]**
Sample size	91,165	91,165	91,165	91,165
**Sibling cohort**				
No Anxiety	0 [Reference]	0 [Reference]	0 [Reference]	0 [Reference]
Week 17	**-34.8 [-58.9, -10.7]**	**-33.2 [-57.3, -9.1]**	-4.5 [-27.4, 18.5]	-4.7 [-27.6, 18.3]
Week 30	**-57.5 [-88, -27]**	**-56.9 [-87.5, -26.3]**	-27.7 [-56.9, 1.5]	-27.9 [-57.2, 1.3]
Both	**-139 [-184.5, -93.4]**	**-134.8 [-180.4, -89.2]**	-33.5 [-77.8, 10.8]	-33.1 [-77.4, 11.2]
Sample size^1^	12,480	12,480	12,480	12,480

^*^Adjusted for smoking and alcohol intake.

^**^Adjusted for smoking, alcohol intake, parity, birth complication, child’s sex and preeclampsia.

^***^Adjusted for smoking, alcohol intake, parity, birth complication, child’s sex, preeclampsia, maternal age, education and marital status.

^1^ Number of sibling “pairs”.

Sibling-comparison analyses found maternal anxiety to be associated with low birthweight when reported in only the 17^th^ gestational week and in only the 30^th^ gestational week. Children exposed to maternal anxiety as reported in both the 17^th^ and 30^th^ gestational weeks were at the highest risk of low birthweight, even after shared-family confounding was adjusted for. This association remained robust after adjusting for smoking and alcohol intake during pregnancy. However, the association was no longer significant when the birth-related and socio-demographic variables were controlled for.

### Regression analyses for prenatal maternal anxiety and gestational age

**Fig. 3:**
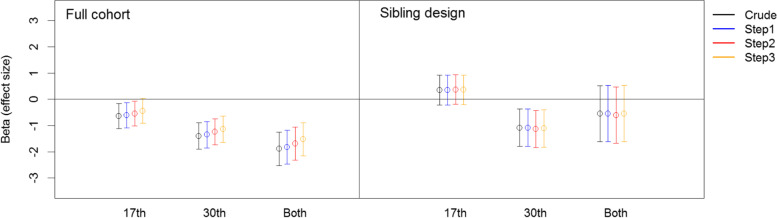
Effect of maternal anxiety on gestational age. Crude: no adjustment; Step1: adjusted for smoking and alcohol intake; Step2: Step1 + parity, birth complication, child’s sex and preeclampsia; Step3: Step1 + Step2 + maternal age, education and marital status

We also examined the association between maternal anxiety and gestation (Table 4 and Fig. 3). In the full cohort, a stronger association was found for those reporting symptoms in the 30^th^ week. Children exposed to maternal anxiety prenatally, across both the 17^th^ and 30^th^ gestational weeks, were at higher risk of short gestational age, compared to those not exposed and those exposed only once during pregnancy. This association was only moderately reduced, after adjusting for smoking and alcohol consumption and remained significant. Similarly to the associations for birthweight, these associations was further reduced, but remained robust after adjusting for multiple birth-related and socio-demographic variables in step 2 and 3.

**Table 4:** Effect of maternal anxiety on gestational age

	Crude	Step1^*^	Step2^**^	Step3^***^
	Beta [95% CI]	Beta [95% CI]	Beta [95% CI]	Beta [95% CI]
**Full cohort**				
No Anxiety	0 [Reference]	0 [Reference]	0 [Reference]	0 [Reference]
Week 17	**-0.64 [-1.12, -0.16]**	**-0.61 [-1.09, -0.13]**	**-0.54 [-1.01, -0.07]**	-0.44 [-0.91, 0.04]
Week 30	**-1.4 [-1.9, -0.89]**	**-1.35 [-1.86, -0.84]**	**-1.23 [-1.73, -0.74]**	**-1.14 [-1.64, -0.64]**
Both	**-1.89 [-2.53, -1.25]**	**-1.82 [-2.46, -1.18]**	**-1.69 [-2.32, -1.06]**	**-1.52 [-2.15, -0.89]**
Sample size	91,165	91,165	91,165	91,165
**Sibling cohort**				
No Anxiety	0 [Reference]	0 [Reference]	0 [Reference]	0 [Reference]
Week 17	0.35 [-0.21, 0.92]	0.35 [-0.21, 0.92]	0.37 [-0.19, 0.94]	0.36 [-0.2, 0.93]
Week 30	**-1.08 [-1.79, -0.37]**	**-1.08 [-1.8, -0.37]**	**-1.13 [-1.84, -0.42]**	**-1.11 [-1.82, -0.4]**
Both	-0.54 [-1.61, 0.52]	-0.54 [-1.61, 0.53]	-0.6 [-1.68, 0.47]	-0.54 [-1.62, 0.53]
Sample size^1^	12,480	12,480	12,480	12,480

^*^Adjusted for smoking and alcohol intake.

^**^Adjusted for smoking, alcohol intake, parity, birth complication, child’s sex and preeclampsia.

^***^Adjusted for smoking, alcohol intake, parity, birth complication, child’s sex, preeclampsia, maternal age, education and marital status.

^1^Number of sibling “pairs”.

In the sibling-design, we found no significant associations between maternal anxiety at 17^th^ week only and gestational age. The association between prenatal maternal anxiety at week 30 and gestational age remained in the sibling cohort, adjusting for shared family effects and control variables in all steps.

## Discussion

This study aimed to examine the effect of both the degree and timing of prenatal maternal anxiety on infant birthweight and gestational age. Therefore, the association between no symptoms, light symptoms and severe symptoms of anxiety with adverse birth outcome and gestation was examined. Timing was also accounted for by examining whether symptoms were reported at either or both of the 17^th^ or 30^th^ gestational weeks. The second aim was to test these associations using a sibling comparison design.

### Main finding and interpretation

Infants of mothers reporting severe anxiety symptoms in the 17^th^ and 30th gestational week were more likely to have lower birth weight than infants born to mothers with very light anxiety symptoms or none at all. This association was reduced, but remained, after adjusting for several controls in the full sample. However, in the sibling subsample, once multiple covariates were controlled for, the association was no longer significant. In regard to prenatal anxiety and gestational age, there was no significant change before and after family adjustment. However in the sibling analyses, only maternal anxiety at week 30 in pregnancy was significantly associated with shorter gestational age.

Mean birthweight was lower for infants born to mothers reporting severe symptoms of anxiety, and the lowest birthweight was found for exposure to anxiety symptoms as reported in the 30^th^ gestational week. These findings are also in accordance with a meta-analysis performed by Ding et al. [5], which found that maternal anxiety was associated with an increased probability of low birthweight. However, after we adjusted for shared family factors and multiple covariates, there were no association between maternal anxiety and birthweight. This finding suggests the importance of controlling for multiple confounding.

In regard to prenatal anxiety and gestational age, maternal anxiety at week 30 in pregnancy was significantly associated with shorter gestational age. This finding suggests a timing effect of maternal anxiety. During the third trimester in a pregnancy, the fetus grows rapidly, and these findings suggest that severe symptoms of anxiety later in pregnancy increase the chance of a short gestational age. The findings persisted also in the sibling analyses, following control for multiple covariates; such as smoking and alcohol exposure, birth complications, and the socio-economic status of the mother. However, we did not control previous psychiatric diagnoses or life stressors, which could be influencing maternal anxiety levels. Nevertheless, this finding is in accordance with previous studies examining prenatal anxiety and birth outcomes [5, 14, 15].

There are several hypotheses regarding the influence of maternal anxiety on fetal growth and gestation. One such mechanism involves changes to maternal hypothalamic-pictuary-adrenal (HPA) axis activity [16] That is, it is suggested that maternal anxiety during pregnancy increases the production of stress hormones such as cortisol and catecholamines [17, 18]. Animal models have found these stress hormones to influence uterine blood flow and immunologic functioning, thus potentially increasing risk of shortened gestational length and lower fetal growth [19]. Another potential explanation for this link, could be through higher risks of infections due to stress. Studies suggests that high levels of stress may be linked to lower immune function [20], and increased infections during pregnancy that could shorten gestation [21]

### Strengths and limitations

There are several strengths to this study. First, the prospective nature of this study reflects the direction of effects and enhances the validity of measurements. In addition, the large sample size provided a range of opportunities to adequately control for confounding factors. This enabled examination of the association between maternal anxiety and birthweight and gestation in a stepwise manner. That is, several confounders could be included at different levels, such as those operating prenatally, as well as those related to birthweight and gestation (e.g. birth complications). Contextual factors such as maternal age and education could also be included. A potential limitation in the current paper, however, is that we were not able to control for previous psychiatric disorders, or the use of medications such as SSRIs, which could be influencing maternal anxiety levels. Next, the unique sample included a large sibling population, which allowed adequate control for family effects such as genetic confounding. This design can be helpful for ruling out measurement error due to familial factors, such as shared genetic factors [11]. Thus, a sibling comparison design using a large cohort study can test whether associations differ between children born after subsequent pregnancies to the same mother. A discordant sibling design could also rule out all environmental differences that may vary between families, which can’t be done using a population comparison design [22].

However, although the sibling design can control for familial factors to a certain extent, it is still important to note that associations could still be confounded by unmeasured unshared environmental factors [23]. The women participating with multiple pregnancies might also represent a biased selection: there is a possibility that the first pregnancy influences the second, as has been suggested by Sjölander et al. [24] and Frisell et al. [25]. It should also be noted that important moderators that could influence maternal anxiety have not been examined (for example, gene-environment interactions). In addition, the self-reported maternal anxiety score might include bias because under-reporting of anxiety symptoms was often observed due to the social stigma about mental health problems, and especially a desire to appear healthy for the expected newborns.

## Conclusion

The main effect of exposure to maternal anxiety on birth weight remained after adjusting for multiple confounding in the full cohort, but not after controlling for shared family confounding. However, the association between maternal anxiety in week 30 and gestational age remained after adjusting for multiple confounding and shared family effects. The implication of these findings, suggests that there is a timing effect for maternal anxiety in third trimester.

## Data Availability

The datasets used and/or analyzed during the current study are available from the corresponding author on reasonable request.

## Abbreviations

PTB: Preterm birth
LBW: low birthweight
MoBa: The Mother Father and Child cohort study
MBRN: Medical Borth Registry of Norway
